# The mediating role of psychological detachment between transformational leadership and work thriving among nurses in Chinese tertiary hospitals: a cross-sectional study

**DOI:** 10.3389/fpsyg.2026.1847889

**Published:** 2026-07-09

**Authors:** Yulian Hu, Dexiu Yan, Junhui Xu, Jiayan Chen, Hong Li

**Affiliations:** 1 The International Peace Maternity and Child Health Hospital, School of Medicine, Shanghai Jiao Tong University, Shanghai, China; 2 Shanghai Key Laboratory of Embryo Original Diseases, Shanghai, China; 3 Department of Nursing, Shanghai General Hospital, Shanghai, China

**Keywords:** mediating role, nurses, psychological detachment, resource conservation theory, transformational leadership, work thriving

## Abstract

**Objective:**

This study adopts a cross-sectional design and selects nurses from tertiary hospitals in China as the research subjects. It aims to explore the influencing mechanism of transformational leadership on work thriving, as well as to examine the mediating effect of psychological detachment between the two variables. From the perspective of the Conservation of Resources Theory, a theoretical model framed as “leadership style–psychological detachment–work thriving” is established.

**Methods:**

From November 2025 to January 2026, a convenience sampling method was adopted to recruit participants from two tertiary hospitals in Shanghai. A total of 1,006 nurses were investigated using the Transformational Leadership Scale, the Psychological Detachment subscale of the Recovery Experience Questionnaire, and the Work Thriving Scale. Bootstrap method was employed for hypothesis testing.

**Result:**

Transformational leadership significantly and positively predicted work thriving (β = 0.743, *p* < 0.001). Psychological detachment partially mediated this relationship (indirect effect = 0.048; 95% CI [0.029, 0.070]), with transformational leadership predicting psychological detachment (β = 0.670, *p* < 0.001) and psychological detachment predicting work thriving (β = 0.172, p <0.001).

**Conclusions:**

This study reveals the intrinsic mechanisms through which leadership styles promote work thriving by facilitating the restoration of nurses‘ psychological resources, thereby providing a theoretical foundation for hospital management practices. Based on these findings, it is recommended that hospital administrators focus on three key areas 0.001). Psychological detachment partially mediated this relationshipment, and promoting individual growth among nurses—to cultivate positive leadership behaviors, establish a systematic psychological support system, effectively enhance nurses' psychological detachment, and ultimately achieve mutual flourishing for both the nursing team and the hospital.

## 1 Introduction

As the pillar of China's healthcare service system, the operational efficiency and service quality of tertiary hospitals are directly related to the national health level (Yi, 2021). Tertiary hospitals in China represent the core level of the tiered healthcare delivery system. They are specifically responsible for the diagnosis and treatment of acute, severe and complex diseases, while also undertaking functions such as technological innovation, talent cultivation, and regional support (The Lancet, 2019). Although tertiary hospitals account for a relatively small proportion of all hospitals nationwide, they handle the vast majority of hospital outpatient and inpatient services, indicating a high concentration of resources and care delivery (Zhao et al., 2014). Within this environment, nurses in tertiary hospitals not only face institutional pressures such as performance evaluations, challenges in patient-clinician communication, and difficulties with career advancement, but also commonly experience direct occupational stressors, including heavy workloads, high emotional demands, and frequent shift work (Jin et al., 2025). As a core component of hospital human resources, nurses' working conditions and psychological health are important guarantors of medical quality and patient safety (Cheng et al., 2020). This cumulatively leads to burnout manifestations such as emotional exhaustion and depersonalization (Cao et al., 2025),which not only undermine the physical and mental health of nurses but also constrain sustained improvements in the quality of care and effective organizational performance (Okuhara et al., 2021).

Work thriving, as an integrated concept, emphasizes the ability of organizations and employees to learn, adapt, and grow in a dynamic environment, and is an important indicator of an organisation's sustainable competitiveness (Fainshtein et al., 2024). For hospital organizations, it is important to promote individual nurses' perceptions of prosperity and to stimulate their motivation to learn and work, in order to meet the challenges of rapidly changing medical technologies and increasingly diverse service need (Bond et al., 2025; Moloney et al., 2020). Leadership style is a key influencing factor in the organizational situation. How to alleviate the exhaustion of nurses' resources and foster organizational prosperity (Gomaa, 2024)by shaping a supportive working environment (Alluhaybi et al., 2023) has become an urgent topic in the field of nursing management.

Psychological Detachment refers to the psychological state in which an individual is freed from work-related matters and thoughts during non-work time and is regarded as an important mechanism for recovering from work-related stress (Baktash and Pütz, 2025). In the case of nurses, due to the specificities of the profession (e.g., night shifts (Y. Li et al., 2022), sudden first aid (Mao et al., 2018), there are greater challenges to achieving adequate psychological detachment, and insufficient psychological detachment can further exacerbate the depletion of resources, creating a vicious circle (F. Zhang et al., 2023). Therefore, exploring how, through effective leadership behavior (Majeed and Fatima, 2020). There is significant theoretical and practical value in promoting psychological detachment among nurses and, in turn, examining its mechanisms of transmission for organizational flourishing.

Transformational leadership is a modern leadership style that emerged after trait, behavioral, and contingency theories (Mayberry, 2024). Avolioio Full Range of Leadership Model (Avolio and Bass, 1991) defines transformational leadership as a leadership style in which leaders inspire followers and drive organizational change through exemplary behavior and value-driven guidance. The American Nurses Association defines transformational leadership in the nursing field as field as a “management style that encourages nurses to take innovative and go beyond basic basic job expectations.” In the close working relationship between nurses and nurse managers, transformational leadership is particularly prominent and widely recognized as the most effective leadership model for promoting psychological detachment and professional fulfillment among nurses. The four dimensions of transformational leadership not only directly stimulate nurses' work enthusiasm and innovative behavior but also, through indirect channels of empowerment and emotional support, effectively alleviate occupational burnout, enhance psychological detachment, and increase work engagement, helping nurses maintain vitality and a zeal for learning(Huang et al., 2025). It is precisely this leadership model—based on the principles of positive psychology and focused on empowerment and motivation—that provides the strongest foundation for promoting nurses' psychological resilience and career growth, making transformational leadership an effective option for stimulating sustainable career development in nursing management.

Nurses in hospitals are constantly exposed to a work environment characterized by high emotional demands, blurred boundaries, and intense stress (Wells, 2024). They commonly face the challenge of “struggling to mentally disconnect from work after their shift ends.” This not only depletes their limited psychological resources but also severely hinders the development of work flourishing (Cobb-Clark and Kettlewell, 2021)—that is, the simultaneous experience of vitality and professional growth in the workplace. Although existing research has confirmed that leadership style influences employees' work engagement (Mphaluwa et al., 2025), most studies are limited to direct effects during working hours or traditional mediating variables (such as autonomy(Dias et al., 2022) and perceived support) (Li and Xing, 2025b), overlooking the cross-domain impact of leadership behaviours on nurses' recovery experiences outside of work. In fact, whether leaders respect personal boundaries, allocate tasks reasonably, and minimize non-work intrusions likely directly determines whether nurses can effectively achieve psychological detachment. According to resource conservation theory (COR) (Hobfoll, 2010), adequate psychological detachment can replenish resources depleted by work, thereby fostering work flourishing. However, there is currently a lack of research treating “leadership style to psychological detachment to work flourishing” as a complete mediational chain; particularly in high-pressure, high-stakes environments such as Grade A tertiary hospitals, this mechanism may be further amplified. Therefore, the core motivation of this study is to examine the mediating role of psychological detachment in the relationship between leadership style and nurses' work flourishing. It aims to provide nursing management with an empirical pathway that begins with “adjusting modifiable leadership behaviors” and promotes work flourishing by enhancing psychological detachment, thereby breaking the vicious cycle where nurses “remain entangled with work after hours, preventing resource recovery and hindering sustained professional growth.” Although transformational leadership has been linked to nurse engagement and well-being in prior research, the mechanism through which it influences work thriving, particularly through post-work psychological recovery, remains underexplored in the Chinese healthcare context. To our knowledge, no study has yet tested psychological detachment as a mediator in this relationship among nurses in Chinese tertiary hospitals.

## 2 Literature review and hypothesis formulation

### 2.1 Leadership style and work thriving

Leadership style is a key variable in influencing subordinates' attitudes, behaviors, and team effectiveness (Irianti et al., 2024). In the context of healthcare organizations, where transformational leadership styles are in the spotlight (Wu et al., 2024). Transformational Leadership refers to the enhancement of intrinsic motivation, ethics and self-efficacy of subordinates through inspirational vision, intellectual stimulation, personalized attention and role modeling (Bass and Riggio, 2006; Deng et al., 2023). Research has shown that transformational leadership creates a climate of innovation (Xie et al., 2018) to promote knowledge-sharing among nurses (Anselmann and Mulder, 2020) with proactive, innovative behavior (Bektaş et al., 2025), laying the foundation for the dimensions of learning needed for work to flourish.

Based on the theory of resource conservation, the supportive behavior of leaders (e.g., providing guidance, giving recognition, empowering decision-making) can be considered a valuable work resource (Iddrisu and Mohammed, 2025). This resource helps nurses respond more effectively to the demands of their work (Broetje et al., 2020), reducing the loss of resources (Wilson et al., 2025) and providing surplus resources to invest in learning and development activities (such as key manifestations of work thriving). Therefore, the present study proposes that:

H1: Transformational leaders positively predict work thriving.

### 2.2 The mediating role of psychological detachment

Psychological detachment is a core dimension of the Recovery Experience (RET) of work stress (Olafsen and Bentzen, 2020). According to the job demands-resources framework (Li et al., 2025d), work demands trigger a short-term load on the individual's psychophysiological systems, which, if not adequately recovered from during non-work hours, can lead to chronic fatigue and long-term health problems. Psychological detachment means that the individual is psychologically “out” of work (Pütz, 2025). Stop thinking about work matters, thus providing an opportunity to restore depleted psychological resources (such as attention, emotional energy).

This study hypothesizes that leadership style can significantly influence employees' psychological detachment levels. On the one hand, transformational leaders, by giving meaning to their work and promoting autonomy (Dias et al., 2022), may reduce nurses' feelings of job alienation (Nurmeksela et al., 2025). This makes it easier for them to psychologically “let go” of their work after work. On the other hand, transformational leaders, by empathizing with their employees' stress, can help them “let go” of their work after work (Lin et al., 2020). Advocating work-life balance (Ratinet et al., 2025). Reducing unnecessary out-of-hours work contacts (such as avoiding non-emergency interruptions after hours) creates an environment conducive to psychological detachment for employees.

According to resource conservation theory, psychological detachment is an important process for restoring and enriching resources. When nurses are able to achieve a higher level of psychological detachment, they are better able to replenish the psychological and emotional resources depleted by their work, and thus enter the new workday with more energy, more positive emotions and better cognitive functioning (Li et al., 2025a; Zhang et al., 2023). This restored state of an individual's resources will directly contribute to his or her learning behavior at work (Caniëls et al., 2022) (e.g., actively seeking feedback, acquiring new skills) and energizing experiences (Söderbacka et al., 2022) (such as passionate and energetic), which are the two core dimensions of individual-level work thriving. When most members of a team are thriving, they will converge to form organizational-level thriving.

In summary, positive leadership styles help nurses achieve psychological detachment by providing them with resources and support, and adequate psychological detachment helps them restore and enhance their personal resources, which in turn increases their likelihood of demonstrating learning and vitality at work and ultimately contributes to work thriving. Therefore, this study proposes that:

H2: Psychological detachment mediates the link between transformational leadership and work thriving.

**Figure 1:**
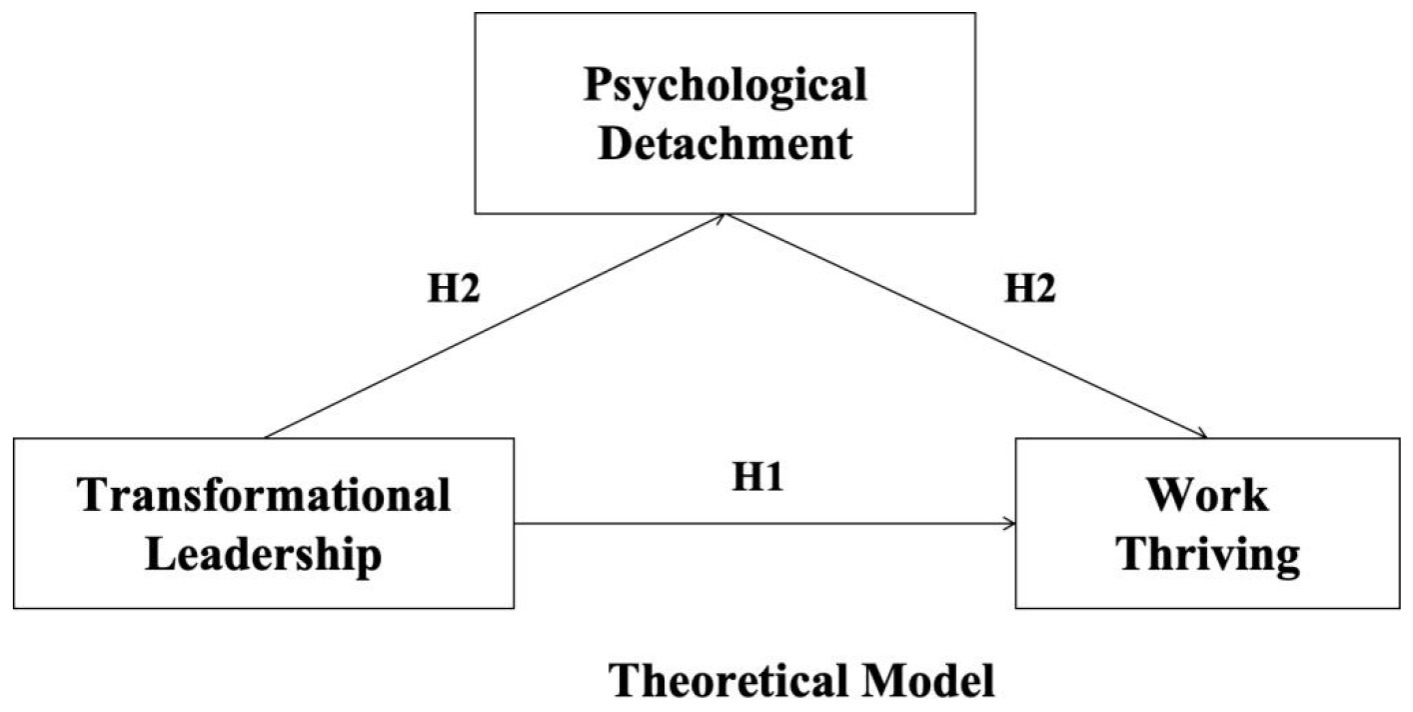
Theoretical model.

Based on the above analysis, the theoretical model constructed in this study is shown in Figure 1.

### 2.3 The research methodology

This study employs a cross-sectional design, and adhered to the STROBE reporting guidelines.

#### 2.3.1 Study population and sampling

Two tertiary hospitals in Shanghai, China, were selected as survey sites between November 2025 and January 2026 using a combination of convenience sampling. The Nursing Department shared the questionnaire in the work group, and the study participants voluntarily completed it. Small gifts were provided to increase the response rate. Patients and/or the public were not involved in the design, conduct, reporting, or dissemination plans of this research.Registered nurses working on the clinical frontline of these hospitals served as the study subjects. Inclusion criteria: (1) qualified to practice as a nurse; (2) working in clinical departments for ≥1 year; (3) informed consent and voluntary participation. Exclusion criteria: (1)Nursing staff in non-clinical positions, such as administration and logistics; (2)Participants who did not complete all items on the questionnaire;(3)Participants who were not at the hospital during the study period (e.g., due to maternity leave, sick leave, etc.)

According to standard guidelines, the sample size should be at least 10-20 times the number of items (Flight and Julious, 2016). This study includes 46 items, so a sample size of 460-920 is required. When conducting mediation analyses using Bootstrap, a sample size of 200 to 500 is typically sufficient for simple mediation models with moderate effect sizes. With the assistance of the hospital's Nursing Department, a total of 1,008 questionnaires were distributed, meeting the sample size requirement. After collecting and excluding invalid questionnaires, such as those with regular answers and excessive missing values, 1,006 valid questionnaires were obtained, with an effective recovery rate of 99.80%. Among the valid samples, 94.7% were females; the average age was 33.99 ± 8.06 years; the average working experience was 12.50 ± 8.58 years; the education level was mainly undergraduate (82.4%); and additional information is shown in Table 1.

**Table 1:** Characteristics of the participants.

Item	Socio-demographic variables	*N* (%)	t/F	*p*
Gender	Female	953 (94.7%)	−3.456	0.001
	Male	53 (5.3%)		
Age (years)	<25	178 (17.7%)	2.439	0.045
	26-30	187 (18.6%)		
	31-35	239 (23.8%)		
	36-40	207 (20.6%)		
	>41	195 (19.4%)		
Work experience (years)	<5	262 (26.0%)	1.636	0.163
	6-10	198 (19.7%)		
	11-15	207 (20.6%)		
	16-20	182 (18.1%)		
	>21	157 (15.6%)		
Department	Internal medicine	84 (8.3%)	7.396	<0.001
	Surgery	144 (14.3%)		
	Gynecology and obstetrics	254 (25.2%)		
	Emergency/ICU	144 (14.3%)		
	Others	380 (37.8%)		
Employment form	Formally employed nurse	528 (52.5%)	1.082	0.339
	Personnel agency	209 (20.8%)		
	Labor dispatch	269 (26.7%)		
Educational level	Diploma and below	177 (17.6%)	2.204	0.028
	Bachelor degree and above	829 (82.4%)		
Job title	Nurse	187 (18.6%)	6.481	0.002
	Primary nurse	586 (58.3%)		
	Intermediate nurse and above	233 (23.2%)		
Marital statu	Single, divorced or widowed	382 (38.0%)	−0.629	0.529
	Married	624 (62.0%)		
Monthly night shift	0 time	300 (29.8%)	8.161	<0.001
	1–5 times	369 (36.7%)		
	>5 times	337 (33.5%)		

The t/F value is used to test for differences in mean job satisfaction levels among nurses with different demographic characteristics

#### 2.3.2 Research tools

All the scales were adopted from mature domestic and international scales through the translation-back-translation procedure and reviewed by experts in nursing management and psychology to ensure content validity. The specific scales are as follows:

##### 2.3.2.1 Transformational Leadership Scale

Adopting the Chinese version of the Transformational Leadership Scale revised by Li Chaoping, (2005). It was used to assess nurses “perceptions of their leaders” transformational leadership style. The scale consists of 26 items, including four dimensions: moral example, visionary inspiration, leadership charisma, and personalized care, and a higher total score indicates a higher level of transformational leadership. The Cronbach's alpha coefficient of this scale was 0.991, and the Cronbach's alpha coefficients of each dimension were 0.972, 0.985, 0.985, and 0.986, respectively.

##### 2.3.2.2 Psychological Detachment Scale

Using the Psychological Detachment Scale of the Recovery Experience Scale developed by Sonnentag and Fritz (2007); Dezhi et al. (2018). The total number of entries was 4 (e.g., “After work, I can leave my work behind for a while”). The Cronbach's alpha coefficient in this study was 0.876.

##### 2.3.2.3 Work Thriving Scale

The work thriving scale compiled by Porath et al. (2012) and translated by Yi and Wenwen (2013). There are 10 items in total, including two dimensions: Learning (5 items, e.g., “I continue to learn new things”) and Vitality (5 items, e.g., “I feel full of energy”), with each item scored from “Not at all” to “Completely meet”, “Not at all” = 1 point, “Completely meet” = 7 points, and “Not at all” = 1 point, “Completely meet” = 7 points. Completely do not meet“ to ”fully meet“ in order to score, ”completely do not meet“ = 1 point, ”fully meet“ = 7 points, the total score and the degree of thriving work. The total score is proportional to the degree of work thriving. The Cronbach's alpha coefficient of the total scale in this study was 0.857, and the learning and vitality sub-dimensions were 0.765 and 0.778, respectively.

##### 2.3.2.4 Control variables

Based on the literature, demographic variables that could have an impact on the main variables were used as control variables, including gender, age, years of service, education, type of department (categorized into high-intensity departments, such as emergency/ICU/operating theater, and general departments) and frequency of night shifts per month.

#### 2.3.3 Methods of analyzing data

Descriptive statistics, common method bias test using SPSS 26.0 (Kock et al., 2021) (Harman one-way method), correlation analysis, and reliability test of the scale. The mediation effect was tested using the bias-corrected non-parametric percentile Bootstrap method with 5,000 replications, and 95% confidence intervals (CIs) were calculated. In statistics, 5,000 bootstrap repetitions are considered the empirical equilibrium point (Johnston and Faulkner, 2021). This is primarily done to control Monte Carlo error (reducing it from 3.2% with 1,000 repetitions to 1.4%), while also balancing computational efficiency. The mediation effect was deemed significant if the CIs did not contain zero. The proportion of the total effect accounted for by the mediating effect of psychological detachment was 6.5% (proportion mediated = 0.048/0.743 ≈ 6.5%).

## 3 Results

### 3.1 Common method bias and validation factor analysis

The results of the Harman one-way test showed that the first factor explained 28.7 per cent of the variance (<40 per cent of the critical value), indicating that common method bias was not serious. The latent method factor approach (Williams and McGonagle, 2016)results of the validated factor analysis showed that the fit indices (χ^2^/df = 2.41, RMSEA = 0.051, CFI = 0.95, TLI = 0.94) of the three-factor model (transformational leadership, psychological detachment, and work thriving) were significantly better than those of the other combined factorial models, which indicated that the variables of the study had good discriminant validity.

### 3.2 Descriptive statistics and correlation analysis

The mean, standard deviation and Pearson correlation coefficients for each variable are shown in Table 2. Transformational Leadership: a significant positive correlation between psychological detachment and work thriving (r-values ranging from 0.195 to 0.572, *p* < 0.01) provides initial support for subsequent hypothesis testing.Multicollinearity diagnostics (Kim, 2019) show that the VIF values of each dimension range between 5.29 and 9.61, indicating that there is no collinearity problem among the dimensions.

**Table 2:** Average and Pearson correlation coefficients.

Item	Average	Moral example	Visionary inspiration	Personalized care	Leadership charisma	Transformational leadership	Psychological detachment	Learning	Vitality	Work thriving
Moral example	4.41 ± 0.70	1								
Visionary inspiration	4.38 ± 0.73	0.879^**^	1							
Personalized care	4.36 ± 0.74	0.862^**^	0.932^**^	1						
Leadership charisma	4.39 ± 0.72	0.861^**^	0.873^**^	0.894^**^	1					
Transformational leadership	4.39 ± 0.69	0.950^**^	0.962^**^	0.962^**^	0.946^**^	1				
Psychological detachment	3.11 ± 0.98	0.173^**^	0.186^**^	0.213^**^	0.175^**^	0.195^**^	1			
Learning	5.49 ± 0.90	0.524^**^	0.520^**^	0.502^**^	0.518^**^	0.541^**^	0.164^**^	1		
Vitality	4.88 ± 1.06	0.491^**^	0.504^**^	0.482^**^	0.468^**^	0.510^**^	0.355^**^	0.677^**^	1	
Work thriving	5.18 ± 0.90	0.552^**^	0.558^**^	0.536^**^	0.536^**^	0.572^**^	0.292^**^	0.901^**^	0.929^**^	1

^**^At the 0.01 scale (two-tailed), the correlation was significant.

### 3.3 Hypothesis testing

#### 3.3.1 Direct effects test

After controlling for demographic variables, structural equation modeling was used to test direct effects. The direct-effects model fit well: χ^2^/df = 2.68, RMSEA = 0.055, CFI = 0.94, TLI = 0.93. Path analyses showed that transformational leadership (β = 0.743, *p* < 0.001) had a significant positive effect on work thriving, and hypotheses H1 were supported.

#### 3.3.2 Mediated effects test

The mediator variable, psychological detachment, was added to the direct effects model, and the full model was constructed. The fit indices of the full model were good: χ^2^/df = 2.52, RMSEA = 0.053, CFI = 0.94, TLI = 0.93. The path coefficients showed that:

1. Transformational Leadership (β = 0.670, *p* < 0.001) significantly and positively predicted psychological detachment.

2. Psychological detachment significantly and positively predicted work thriving (β = 0.172, *p* < 0.001).

3. When psychological detachment was included, the indirect effect of transformational leadership (β = 0.048, *p* < 0.001) on work thriving remained significant, but the coefficient decreased, suggesting that partial mediation may exist.

The results of the Bootstrap mediation test (see Table 3) confirmed that the indirect effect of psychological detachment on the relationship between transformational leadership and work thriving was 0.048, with a 95% CI of [0.029, 0.070] and did not include 0, and hypotheses H2 were supported. Therefore, the partial mediation effect of psychological detachment was significant, and the study's hypothesis was supported.

**Table 3:** Analysis of the mediating effect of psychological detachment between transformative leadership and work thriving.

Item	Effect	se	*t*	*p*	LLCI	ULCI
Total effect of transformative leadership-work thriving	0.743	0.338	22.087	0.000	0.681	0.814
Direct effects of transformative leadership-work thriving	0.670	0.037	20.798	0.000	0.634	0.766
Direct effects of psychological detachment-work thriving	0.172	0.237	7.279	0.000	0.126	0.219
Indirect effects of transformative leadership-work thriving	0.048	0.010	/	/	0.029	0.070

## 4 Discussion

### 4.1 The role of leadership style in work thriving

The present study found that transformational leadership was effective in predicting perceived work thriving among nurses in a tertiary care hospital, consistent with previous studies in corporate organizations (Agyabeng, 2025) and extending to high-stress health care situations. Transformational leaders have stimulated intrinsic motivation in nurses through “meaning-giving” (e.g., linking daily nursing tasks to the noble mission of saving lives and promoting health) (B. Zhang et al., 2025) and enthusiasm for learning (Masood and Afsar, 2017). This makes them more willing to take the initiative to update their knowledge and skills to cope with complex conditions. In addition, transformational leaders create a safe and supportive work environment through “empowerment and caring” (e.g., listening to nurses' difficulties, delegating authority, and attending to their family needs) (Hasan et al., 2023; Zusman et al., 2025). This enhanced nurses' sense of psychological security and belonging, which made them more courageous in expressing their views, attempting to innovate in nursing practice, and demonstrating greater vigor in their work. Transformational leadership style provides crucial contextual resources for work prosperity through the two paths of “stimulating ideals” and “reinforcing support” respectively.

### 4.2 Key mediating mechanisms for psychological detachment

The most important finding of this study is the role of psychological detachment in bridging the gap between leadership style and work thriving. The results support the inference from resource preservation theory that positive leadership behaviors, as a valuable social support resource, can help nurses better psychologically “isolate” themselves from work stressors (Lobato et al., 2024; Sariköse et al., 2026). For example, transformational leaders who are skilled in personalized care can detect emotional exhaustion among nurses and advise them on how to adjust (Larson et al., 2023). Transformational leaders who genuinely care about their subordinates‘ wellbeing will proactively promote a team culture that respects time off (Shi et al., 2023). These behaviors contribute directly or indirectly to the quality of nurses' psychological detachment during non-work time.

High-quality psychological detachment leads to a fuller recovery of psychological resources. Nurses who have been adequately restored (Li et al., 2025c). Return to work with greater cognitive (e.g., attention, judgement) and affective (e.g., motivation, empathy) resources. This makes them more able and willing to engage in “learning” behaviour (S. Zhang et al., 2022) (e.g., studying difficult cases, learning to operate new equipment), and they are also more likely to experience a sense of “vigor”(Kadosh and Rozani, 2025) (e.g., energetic and willing to collaborate). This learning and dynamism at the individual level converge into innovation and a positive atmosphere at the team and unit levels, which ultimately contribute to the prosperity of the entire nursing unit and the hospital organization (Wang et al., 2024). The discovery of the mediation effect suggests that managers need not only “push” (direct motivation) but also “pull” (indirectly through recovery) to promote prosperity. The discovery of the intermediary effect suggests to managers that promoting work prosperity requires not only “push” (direct stimulation) but also “pull” (indirect enhancement through restoration), and that psychological detachment is an indispensable part of the latter.

The findings of this study, which indicate that psychological detachment mediates the relationship between leadership style and work thriving, are similar to those showing that psychological detachment partially mediates the relationship between emotional labor and job engagement among emergency department nurses (β = 0.054) by Guowen et al., 2020. The fundamental reason is that the high emotional workload, high moral pressure, lack of autonomy in work rhythms, and dual physiological and psychological exhaustion experienced by nurses weaken the mediating efficacy of “psychological detachment,” a relatively “cognitive-restorative” mechanism. The findings indicate a lower mediating effect of psychological detachment on work-related connectedness behaviors and time conflict among corporate employees (β = 0.22, *P* < 0.01) by Hongyu et al., 2014. Possible reasons include: First, differences in occupational contexts. Nurses face high emotional demands and moral pressure rather than mere time pressure; psychological detachment is more effective at alleviating “cognitive rumination” but has limited efficacy in addressing “emotional rumination.” Second, differences in the nature of the dependent variables. Work engagement is a positive psychological state dominated by the resource-gain pathway, whereas time conflict is a negative conflict perception dominated by the resource-depletion pathway; the latter is more directly explained by insufficient psychological detachment. Third, the highly permeable boundaries and shift work schedules(B. Li et al., 2026) characteristic of nursing make it far more difficult for nurses to practice psychological detachment than for typical corporate employees, thereby weakening its buffering effect. Future research could further compare the distinct roles of psychological detachment in emotional versus cognitive work stress.

### 4.3 Practical insights

#### 4.3.1 Leadership development

To optimize nursing management in tertiary hospitals, it is essential to systematically incorporate the core elements of transformational leadership into the training and assessment systems for managers such as chief nurses and directors of nursing departments (Kelly et al., 2014). Specifically, through workshops, case studies, and coaching, efforts should focus on enhancing abilities in visionary motivation, personalized care, and the empowerment of subordinates. As a result, these measures will help shape a supportive management style.

#### 4.3.2 Organizational culture

A supportive organizational culture for psychological detachment should be built at multiple levels. Institutionally, this means allocating scientific staff, optimizing shift scheduling, and prohibiting non-emergency work during breaks (Scott-Marshall, 2024). Leaders should promote work-life balance and encourage full relaxation during breaks. Staff assistance programmes and psychological skills training, such as stress reduction and time management, should be introduced to help nurses master active detachment techniques (Talebiazar et al., 2025).

#### 4.3.3 Individual capacity building

Managers should create learning opportunities through mini-lectures, case discussions, and supporting further training (Bekaert, 2025). Establishing awards for innovative proposals and effective teams recognizes nurses” contributions and links individual prosperity with organizational development, stimulating the team's vitality and momentum.

Based on the study's conclusions, to optimize nursing management in tertiary hospitals, it is recommended that three aspects—leadership development, cultural environment construction, and individual prosperity—be promoted together. First, transformational leadership elements should be systematically incorporated into manager training and assessment, including for chief nurses and directors of nursing.

### 4.4 Study limitations and future directions

This study has certain limitations. First, the cross-sectional design does not allow for strict inference of causal relationships among variables; future studies could employ longitudinal designs or diary methods to further validate these findings. Second, all data were derived from nurses' self-reports and were subject to leadership influence; although statistical tests indicated that common-method bias was not severe, future studies could incorporate multi-source data, such as evaluations by supervisors and colleagues. Third, the sample primarily consists of nurses from comprehensive Grade A tertiary hospitals, and the generalizability of the findings to specialty hospitals or primary care facilities remains to be tested. Future research could further explore the mediating roles of other recovery experiences (such as a sense of mastery and relaxation) or introduce moderating variables such as workload and team psychological safety to more comprehensively reveal the boundary conditions and complex mechanisms through which leadership style influences work thriving.

## 5 Conclusion

Based on resource preservation theory, this study demonstrated that transformational leadership in a tertiary care hospital not only contributes directly to work prosperity but also works indirectly by enhancing nurses' psychological detachment. The results of this study emphasize the importance of resource recovery and nurses' psychological experiences outside the workplace for the long-term health of the organization in a high-stress healthcare work environment. Hospital administrators should implement a dual-path strategy: on the one hand, they should focus on cultivating positive leadership to provide nurses with adequate work resources; on the other hand, they should actively create supportive environments to safeguard and promote nurses' psychological detachment, so as to achieve synergistic prosperity and sustainable development of both individual nurses and the organizational structure of the hospital.

### 5.1 Permission to reuse and copyright

This is an open-access article distributed under the terms of the Creative Commons Attribution

License (CC BY). The use, distribution or reproduction in other forums is permitted, provided the original author(s) and the copyright owner(s) are credited and that the original publication in this journal is cited, in accordance with accepted academic practice. No use, distribution or reproduction is permitted which does not comply with these terms.

## Data Availability

The raw data supporting the conclusions of this article will be made available by the authors, without undue reservation.
